# Antibiotic Treatment of Anaerobic Infections: Uncharted Land in Iran

**Published:** 2019-11

**Authors:** Mohammad Yousef MEMAR, Mina YEKANI, Mohammad AGHAZADEH, Hossein BANNAZADEH BAGHI

**Affiliations:** 1. Immunology Research Center, Tabriz University of Medical Sciences, Tabriz, Iran; 2. Students’ Research Committee, Tabriz University of Medical Sciences, Tabriz, Iran; 3. Infectious and Tropical Diseases Research Center, Tabriz University of Medical Sciences, Tabriz, Iran; 4. Drug Applied Research Center, Tabriz University of Medical Sciences, Tabriz, Iran; 5. Department of Virology, Faculty of Medicine, Tabriz University of Medical Sciences, Tabriz, Iran

## Dear Editor-in-Chief

Antimicrobial resistance among some anaerobic pathogens has increased considerably over the last few decades. Anaerobic bacteria are significant pathogens found in several infections. However, many laboratories are either incapable or unwilling to culture these bacteria except very specific sites and even fewer laboratories detect antimicrobial susceptibility patterns. Therefore, the antibiotic susceptibility pattern of anaerobes remains virtually unclear to laboratorians and clinicians, except for those published surveys performed by small personnel of research centers scattered worldwide or contained in individual case reports. Resistance trends in anaerobes are therefore biased and sometimes anecdotal (1, 2). For this reason, the treatment of anaerobic infections, in many hospital settings, not only starts but remains empirical based on published surveys. However, both the global and local changes in resistance rates over time should be considered to provide optimal treatment for patients.

Susceptibility testing of anaerobes is crucial in severe infections such as those found in the bloodstream and in isolates obtained from normally sterile body sites as well as those non-responsive to empirical therapy. Anaerobes should be considered for susceptibility testing if they are highly virulent pathogens and have un-foreseeable susceptibility patterns (3, 4). Some examples of these include the Gram-negative species *Bacteroides* spp., *Prevotella* spp., *Fusobacterium* spp., *Bilophila wadsworthia,* and *Sutterella wadsworthensis* as well as the Gram-positive *Clostridium* spp. (5).

A good understanding of antibiotic susceptibility patterns is essential for the treatment of anaerobic infections. However, as of now there is no comprehensive published survey on antibiotic susceptibility patterns of most anaerobes from Iran. Some partial studies, including small sizes of specimens, reported of having high frequencies of antibiotic resistance among some anaerobes from Iran. Consequently for the treatment and control of anaerobic infections, comprehensive investigations based on confirmed guidelines and standardized methods are essential and unavoidable. These surveys are a primary step for the adjustment of surveillance programs. This is why factors such as the accessibility of antibiotics, financial issues and the epidemiology of anaerobic infections should be respected in order to receive appropriate and practicable results.
